# Does the Application of ICTs Improve the Efficiency of Agricultural Carbon Reduction? Evidence from Broadband Adoption in Rural China

**DOI:** 10.3390/ijerph19137844

**Published:** 2022-06-26

**Authors:** Pan Rao, Xiaojin Liu, Shubin Zhu, Xiaolan Kang, Xinglei Zhao, Fangting Xie

**Affiliations:** 1 School of Management and Economics, Jiangxi Agricultural University, Nanchang 330045, China; raopan@stu.jxau.edu.cn (P.R.); xiaojinjin06@jxau.edu.cn (X.L.); zxl0212021226@stu.jxau.edu.cn (X.Z.); 2 Institute of Rural Development, Jiangxi Agricultural University, Nanchang 330045, China; kxl5364@jxau.edu.cn; 3 College of Economics and Management, Zhejiang A&F University, Hangzhou 311300, China; 4 Research Academy for Rural Revitalization of Zhejiang Province, Zhejiang A&F University, Hangzhou 311300, China

**Keywords:** agricultural carbon reduction efficiency, broadband adoption, rural China, moderating effects

## Abstract

Based on the Environmental Kuznets Curve (EKC) hypothesis, this paper examines whether rural broadband adoption affects agricultural carbon reduction efficiency (ACRE), using panel data from 30 Chinese provinces from 2011 to 2019. This paper achieves a measurement of ACRE by taking the carbon sink of agricultural as one of the desired outputs and using a Slacks-Based Measure (SBM) model and the global Malmquist–Luenberger (GML) index. The results show that: (1) Rural broadband adoption has a positive effect on ACRE. The relationship between the income of rural residents and ACRE was an inverted U-shaped, which confirms the EKC hypothesis. (2) Land transfer has a significant promoting effect on the relationship between rural broadband adoption and ACRE. When the land transfer rate is high, the positive effect of broadband adoption is obvious. (3) The positive effect of broadband adoption on ACRE was more obvious when farmers invested more in production equipment, that is to say, it has a significant positive moderating effect. As farmers in many developing countries suffer from increasingly frequent and severe extreme weather events, we believe that the results of this study also have implications for the implementation of agricultural carbon reduction and smart agricultural equipment roll-out in many countries.

## 1. Introduction

The sustainable development of agricultural systems has become a more urgent issue in the context of global challenges such as the COVID-19 pandemic and food insecurity. Climate change has substantial impacts on water balance, affecting the surrounding industries, agriculture, and other economic sectors [1,2]. Carbon dioxide (CO_2_) is the main gas causing climate change and the greenhouse effect [3]. Therefore, reducing CO_2_ emissions and promoting sustainable agriculture have become major goals for global development. In the past, due to a large population and limited arable land, China’s agricultural industry focused strongly on extensive farming. China’s high-input, high-consumption, and high-pollution agricultural development model has resulted in significant pressure on resources and the environment, threatening the sustainability of agriculture [4]. In order to overcome these challenges, it is essential to implement timely strategies for improving the efficiency of agricultural carbon reduction [5,6]. The progress of digital technology provides a new way of breaking through the bottleneck of current agricultural development and finding a green development strategy. Integrating data and information elements into agricultural systems can help optimize factor allocation and facilitate the efficient and coordinated development of agricultural systems [7,8]. Digital agriculture in many developed countries is based on the concept of green and sustainable development. For example, according to the precipitation, soil fertility, and climate, Australia ensures efficient and green agricultural production through agricultural information monitoring and agricultural decision support systems [9]. Germany’s large agricultural machinery equipped with information technology can carry out all kinds of farm operations, such as precise sowing, fertilization, weeding, and harvesting. The adoption of these technologies can achieve quantitative fertilization and spraying in different places within the same plot to ensure the efficient utilization of chemicals and fertilizer while avoiding environmental pollution [10]. As a developing country, China, like many developed countries, vigorously develops digital agriculture and actively promotes the application of information and communication technologies (ICTs) in green agriculture. At present, with the vigorous implementation of the “Digital Villages” strategy, the supply of ICTs in rural areas is increasing rapidly. According to the *Communication Industry Statistical Bulletin*, by the end of December 2019, the net increase in rural broadband users was 17.36 million, which was 14.8% higher than the previous year. This growth rate was 6.3 percentage points higher compared to urban broadband users in the same year. Furthermore, digital technology is spreading rapidly into agriculture. Agricultural informatization has become an important power source to promote the high-quality development of China’s agriculture. Therefore, embedding ICTs as external technology and studying their impact on agricultural carbon reduction efficiency (ACRE) is key to testing the actual effect of the “Digital Villages” project in China.

Three core issues are being discussed in existing literature on agricultural carbon reduction and its relationship with ICTs. The first core issue is of updating the measurement index and method for ACRE. Carbon reduction efficiency (CRE) is a popular concept in industrial and urban economies and is often used to measure the gap between the actual CO_2_ emissions generated by manufacturing or other human activities and the optimal CO_2_ emissions [11,12]. According to this concept, ACRE is defined as the ratio of the theoretical minimum CO_2_ emissions of agricultural production activities to the actual CO_2_ emissions under fixed input-output conditions. ACRE directly reflects the effect of regional agricultural CO_2_ emissions and indirectly reflects the potential of regional agricultural CO_2_ emissions. The literature on ACRE measurement is mainly divided into three parts: One is the selection of the measurement model, another is the selection of measurement index, and the final one is the selection of measurement indicators. First, for model selection, the Solow residual, Algebraic Index, and Stochastic Frontier Approach (SFA) can only fit the production process of one kind of output. In contrast, the Data Envelopment Analysis (DEA) method does not need to set a specific form of production function, but the traditional DEA model has one distinct limitation: non-effective units can only rely on radial improvement to reach the frontier; that is, increasing or decreasing the input and output in the same proportion. In order to overcome this limitation of the traditional DEA model, Tone [13] proposed the Slacks-Based Measure (SBM) model, which involved the addition of undesirable outputs, such that the output and input can be adjusted in non-radial directions by non-effective units. Second, regarding the choice of index, there are three main kinds of productivity index: the Malmquist non-parametric linear programming algorithm; the Luenberger productivity index further developed by Chambers et al. [14]; and the modified version by Chung and Fare [15] which included undesired output in the Malmquist–Luenberger index. Unfortunately, these indexes all have problems of intransitivity and infeasible solutions. A study by Oh [16] showed that the method of global reference can solve these problems, and the GML (Global Malmquist–Luenberger) index, constructed by global reference, can measure ACRE more accurately. Third, in terms of the input indicators, these mainly include agricultural labor force, farmland, machinery, chemical fertilizer, pesticides, irrigation, and other indicators. Desired output refers to agricultural output while undesired output indicates agricultural CO_2_ emissions [17,18,19].

The second core issue is the identification of the main factors driving agricultural carbon reduction. At present, the literature mainly focuses on the sources of agricultural CO_2_ emissions and changes in food demand. First, regarding CO_2_ emissions, micro measures to combat this issue focus on changes in land use type and the return of grassland and farmland to forest [20], as well as chemical application and residue, agricultural waste treatment, and livestock and poultry manure management [21,22]. Agricultural production structure and regional economic development are considered to have a positive impact on agricultural CO_2_ emissions reduction at the macro level [23]. It was confirmed that agroecological efficiency was also affected by the agricultural industrial structure characterized by the proportion of the sown area of food crops to the total sown area of crops. Chen et al. [24] found Kuznets Curve characteristics between agricultural industrial agglomeration and agricultural carbon efficiency. Second, with respect to food demand, strategies may include adjusting the diet structure, reducing the proportion of meat intake, replacing animal protein with plant protein, and reducing food loss and waste [25,26]. Empirical studies have found that the price mechanism and the promotion and education of healthy eating and on-demand consumption can effectively change consumers’ behavioral preferences and influence the structure and quantity of food demand [27,28], thus promoting agricultural carbon reduction.

The third core issue is the relationship between ICTs and agricultural carbon reduction (sustainable agriculture). In practice and theory, it is indisputable that ICTs directly affect the productivity of agriculture [29,30]. In the field of agricultural informatization, ICTs are expected to enhance the abilities of farmers, with the use of diverse tools to obtain all kinds of information. For example, mobile messaging applications can support instant access to market information, weather data, production advice, and financial services-related information [31]. By observing the development of green agriculture in Serbia and all EU countries, some scholars believed that the use of precision agriculture, automatic management technology systems, and geographic information systems hage led to the rational use of inputs, thus reducing the adverse impact on the environment [32]. However, others hold different views. Big data can improve the economic and environmental performance of agriculture but may threaten the sustainability of agri-food systems, especially by exacerbating the gap between small-scale and large-scale farming [33]. Precision agriculture supported by broadband internet is widely considered to have more environmental benefits than drawbacks. For example, compared with traditional agriculture, the environmental benefits of precision agriculture include the possibility of reducing agricultural greenhouse gas emissions [34,35]. Unfortunately, the policy-related mechanisms of technology adoption required for green agriculture are rarely explored.

The above three core issues for research and progress have important theoretical value and policy implications for analyzing the impact of ICTs on ACRE. Indeed, broadband is the main infrastructure for carrying information, and household broadband penetration within a region is often used to represent ICTs development level [36,37,38]. Our paper defines broadband adoption as an ICTs application. As China’s rural broadband access becomes increasingly common and more individuals routinely go online, it provides a good foundation for the operation of agricultural informatization production equipment. Moreover, under the role of information technology, facility agriculture can better realize the precision management of farmland. This can be attributed to the increased accessibility to precision agriculture that is provided by broadband adoption. The research status in this field is as follows. (1) Apart from a few studies [32,39], most studies on agricultural carbon reduction do not consider broadband adoption as an external factor. (2) In terms of research methods, in the studies that considered broadband to reduce carbon emissions, the analysis method were mainly observation and comparison, rather than econometric model analysis. Based on existing research, since agricultural production acts as a carbon sink [40,41], measurement accuracy can be improved by taking the carbon sequestration of agricultural production as one of the indicators of expected output. (3) Few scholars have discussed the moderating effect of land transfer and farmers’ investment in production equipment on ACRE in the context of rural broadband adoption.

This paper argues that there is currently a lack of discussion on the mechanism by which rural broadband adoption affects agricultural carbon reduction, which may dilute its importance in agricultural sustainability. Therefore, in the context of China’s “Digital Villages” project and carbon reduction strategy, this paper uses provincial panel data and econometric modeling from 2011 to 2019 to verify the impact and moderating effect of ICT application on ACRE. The results show that in accordance with the environmental Kuznets Curve (EKC) hypothesis, rural broadband adoption has a positive effect on ACRE. In addition, we also find that higher rates of broadband access are associated with greater improvements in ACRE through increased land transfer rates. With the improvement of informatization, farmers’ investment in production equipment also plays a positive role in ACRE.

Compared with previous studies, the main contributions of this paper are as follows. (1) It confirms the positive impact of rural broadband adoption on ACRE, complementing existing evidence on the influencing factors of ACRE. (2) This paper analyzes the moderating effect of land transfer and farmers’ investment in production equipment on ACRE in the context of rural broadband adoption, and the conclusion has a certain reference value for the promotion of land transfer and smart agricultural equipment decision-making. (3) Our paper takes the carbon sink of agricultural production as one of the desired outputs and uses SBM modeling and the GML index method to measure ACRE more comprehensively.

This paper is organized as follows. After the Introduction, Section 2 is the theoretical analysis and research hypothesis. Then, we describe the research object, data sources, and method in Section 3. Section 4 presents our empirical results and carries out a series of robustness and endogeneity tests. Section 5 concludes the paper.

## 2. Theoretical Analysis and Hypotheses

This study analyzes the EKC hypothesis considering the role of broadband adoption. The nexuses between rural broadband adoption and agricultural carbon reduction are complex. The micro-mechanism of broadband adoption influencing agricultural CO_2_ emission reduction is divided into two strands. The first strand is direct effects. The ultimate goal of new technologies is reflected in the long-term sustainability of agriculture, and broadband adoption provide a foundation for precision agriculture. The use of precision-agriculture-applied automated management technology systems and geographic information systems has led to increased yields, while also reducing the adverse impact on the environment [32]. Environmental benefits of precision agriculture compared to traditional agriculture include the potential to reduce waste from fertilizer and pesticide application, save water [42,43], and mitigate agricultural greenhouse gas emissions [34]. Unfortunately, precision agriculture can only be adopted by farmers who have access to broadband due to the technological requirements [44]. In previous studies, Wang et al. [45] and Ma and Zheng [46] found a significant correlation between Internet use and farmers’ environmental behavior of reducing fertilizers and pesticides.

The second strand is the propagation effect. Residents trust public positive information the most and trust private negative information the least [47]. The spread of positive information, such as environmental protection, has been accelerated by the popularization of broadband adoption. This has promoted the awareness of low-carbon consumption in daily life. The development of broadband has allowed people to quickly access information related to environmental pollution causes and hazards [48]. Smart phones commonly used by farmers can not only improve environmental awareness and regulate farmers’ behavior, but also make more farmers aware of the perceived threat of environmental degradation [49]. In summary, we propose the following hypotheses:

**Hypotheses** **1** **(H1).** *Rural broadband adoption directly promotes the improvement of ACRE*.

The EKC hypothesis illustrates that rising income contributes to pollution but after up to a point, after which pollution decreases. However, pollution changes with income due to scale, composition, and technique effects [50,51]. Some scholars focus on the non-linear effects of economy and income on agricultural CO_2_ emissions. Based on the estimation of agricultural CO_2_ emissions in China from 1991 to 2018, the EKC model is used to conclude that economic and income growth is the main driving factor of agricultural CO_2_ emissions [52]. In summary, we propose the following hypotheses:

**Hypotheses** **2** **(H2).** *The income of rural residents has a non-linear effect on ACRE*.

Theoretically, the mismatch of production materials directly inhibits output and distorts the input decisions of micro subjects, resulting in the loss of environmental welfare [53]. Under the micro-scale efficiency driving mechanism, the development of land transfer market will increase land use to reduce agricultural yield losses [54]. Existing studies have shown that compared with small farmers, big farms with strong operational capacity, rich production experience and a high level of professionalism are more likely to accept and adopt low-carbon agricultural technologies [55]. As we know, land transfer promotes the large-scale management of cultivated land, and then changes the land use, thus regulating agricultural CO_2_ emissions

The widespread development of broadband adaption around 2010 enabled precision agriculture to develop web services resulting in information equipment such as spraying drones and soil temperature sensors [44]. Several barriers exist to adopting precision agriculture technologies, aside from broadband access. These include technical issues for the equipment itself, disconnect or lack of compatibility between the precision agriculture equipment and the farm operation, concerns regarding the misuse of agricultural data, managing the large amounts of data precision agriculture provides, lack of user-friendly designs and interfaces, and high costs of implementation [56]. Hence, we propose the following hypothesis:

**Hypotheses** **3** **(H3).** *Land transfer plays a positive moderating role between broadband adoption and ACRE*.

**Hypotheses** **4** **(H4).** *The investment of farmers in production equipment plays a positive moderating role between broadband adoption and ACRE*.

**Figure 1:**
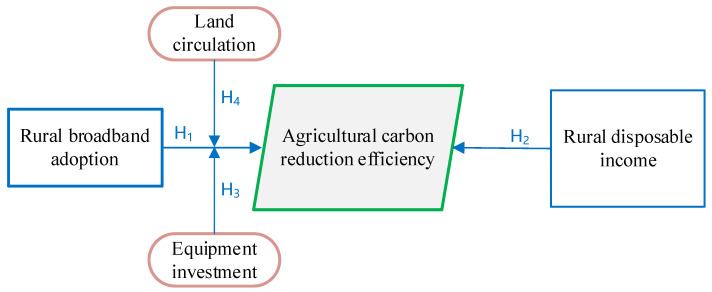
Theoretical model of this study.

Based on the above research hypothesis, the theoretical analysis framework of this study is obtained (Figure 1).

## 3. Materials and Methods

### 3.1. Data and Samples

In order to investigate the direct impact of rural broadband adoption on ACRE and its related moderating effect, this paper uses panel data of 30 provinces (excluding Tibet, Hong Kong, Macao, and Taiwan) from 2011 to 2019. First, the dependent variable is ACRE, which is measured using input and output data. Second, the core independent variable is the rural broadband adoption ratio (*Broadband*). Finally, the control variables and moderator variables include rural disposable income (*Income*), rural disposable income squared (*Income square*), operation scale (*Scale*), agricultural economic status (*Status)*, industrial added value (*Industrialization*), the ratio of disaster (*Damage*), land transfer (*Ltr*), and equipment investment (*Equipment*). The above data mainly come from the *China Rural Statistical Yearbook*, *China Statistical Yearbook*, *China Agricultural Machinery Industry statistical Yearbook*, and *China Environmental Statistical Yearbook*.

### 3.2. Variables

#### 3.2.1. Dependent Variable

By definition, ACRE should be measured according to the actual level of agricultural carbon emissions and the theoretical optimal CO_2_ emissions. Based on existing research methods, this paper adopts the SBM model and GML (Global Malmquist–Luenberger) index method to calculate ACRE [57,58,59,60,61]. The measurement index system for the ACRE is as follows (Table 1). Labor input, land input, machinery input, fertilizer input, irrigation input, and electricity input are applied as input indicators. Referring to Liu et al. [23], the total output value of agriculture, forestry, animal husbandry and fishery, and the CO_2_ sink of agricultural production are taken as desirable outputs, and the undesirable output is CO_2_ emissions generated in the process of agricultural planting.

The reasons for choosing this approach are as follows. First, the GML index method can simulate multiple inputs and outputs simultaneously to accurately measure ACRE. Second, the SBM model can measure efficiency from multiple angles, evaluate the impact of non-zero input and non-zero output relaxation, and comprehensively measure ACRE. Finally, in view of the above advantages, the SBM model and GML index method are suitable for estimating the efficiency of agricultural carbon emissions reduction.

Referring to Meng and Qu [62], according to the global comparison strategy, each province in each year is regarded as a decision-making unit (DMU). Suppose each province has m inputs, $r_{1}$ desirable outputs, and $r_{2}$ undesirable outputs. Then, under VRS (Variable Returns to Scale), the general form of the SBM model can be constructed as follows:

(1) $$\min\;\rho =\frac{\frac{1}{m}(\sum_{i=1}^{m}\frac{\bar{x}}{x_{ik}})}{\frac{1}{r_{1}+r_{2}}(\sum_{w=1}^{r_{1}}\frac{{\bar{y}}_{w}}{y_{wk}}+\sum_{w=1}^{r_{2}}\frac{{\bar{p}}_{u}}{p_{uk}}}\;s.t.\{\begin{array}{c} {\bar{x}}_{i}\geq \overset{n}{\underset{j=1,\neq k}{\sum }}x_{ij}\lambda_{j},i=1,2,\ldots ,m;\\ {\bar{y}}_{w}\geq \overset{n}{\underset{j=1,\neq k}{\sum }}y_{wj}\lambda_{j},i=1,2,\ldots ,r_{1};\\ {\bar{p}}_{u}\geq \overset{n}{\underset{j=1,\neq k}{\sum }}p_{uj}\lambda_{j},i=1,2,\ldots ,r_{2};\\ \lambda_{j}\geq 0,{\bar{x}}_{i}\geq x_{ik},{\bar{y}}_{w}\geq y_{wk},{\bar{p}}_{u}\geq p_{uk};j=1,2,\ldots ,n\left(j\neq k\right) \end{array}$$

In Equation (1), ρ is the objective function. $x_{ij}$, $y_{wj}$, and $p_{uj}$ are the relaxation variable of input, desirable output, and undesirable output, respectively, and λ is their weight. According to the definition of agricultural CO_2_ emissions reduction (the ratio of possible minimum carbon emission from agricultural production to actual carbon emission under fixed input and fixed economic output), based on the calculation of the SBM model, the GML index is determined as follows:

(2) $$GML_{k}^{t,t+1}=\frac{1+D^{G}\left(x_{k}^{t},y_{k}^{t},p_{k}^{t}\right)}{1+D^{G}\left(x_{k}^{t+1},y_{k}^{t+1},p_{k}^{t+1}\right)}\;=\frac{1+D^{t}\left(x_{k}^{t},y_{k}^{t},p_{k}^{t}\right)}{1+D^{t+1}\left(x_{k}^{t+1},y_{k}^{t+1},p_{k}^{t+1}\right)}\times \left[\frac{1+D^{G}\left(x_{k}^{t},y_{k}^{t},p_{k}^{t}\right)}{1+D^{t}\left(x_{k}^{t},y_{k}^{t},p_{k}^{t}\right)}\times \frac{1+D^{t+1}\left(x_{k}^{t+1},y_{k}^{t+1},p_{k}^{t+1}\right)}{1+D^{G}\left(x_{k}^{t+1},y_{k}^{t+1},p_{k}^{t+1}\right)}\right]\;=EC_{k}^{t,t+1}\times TC_{k}^{t,t+1}$$

In Equation (2), $GML_{k}^{t,t+1}$ represents the two stages of the change in CO_2_ reduction efficiency in each province. $D^{G}$ represents a global directional distance function dependent on production possibilities. $EC_{k}^{t,t+1}$ represents the change in technical efficiency; $TC_{k}^{t,t+1}$ represents the technical progress index. A smaller GML value indicates a greater deviation between the actual agricultural CO_2_ emissions and the minimum possible CO_2_ emissions, and therefore implies greater redundancy in agricultural carbon emissions and lower efficiency of carbon emissions reduction.

#### 3.2.2. Main Independent Variable

In the Introduction, the impact of ICT application on agricultural carbon reduction was described in detail. Broadband penetration, as a measure of ICT development, is usually measured by the proportion of broadband connections available [38,63] Therefore, this paper uses the proportion of actual rural broadband users out of the total regional users to measure rural broadband adoption as the main independent variable, expressed by *Broadband*. In the robustness test, the penetration rate of household computers in rural households, i.e., the ownership of household computers per 100 households, is selected for reference Zhang [64] and is represented by *Computer*. This variable is selected because rural households need Internet terminal equipment to install fixed broadband.

#### 3.2.3. Other Variables

The factors affecting ACRE are complex, so we also added control variables. Some previous studies have focused on the non-linear effects of the economy and income on agricultural CO_2_ emissions. The EKC model estimates that economic growth is the main driving factor of agricultural CO_2_ emissions in China from 1991 to 2018 [52]. Hence, we choose rural per capita disposable income and income squared as control variables. Previous studies found that the extent of input of agricultural CO_2_ sources (such as fertilizers and chemicals) showed an obvious U-shaped trend with increasing farmland size [65]. Therefore, the sowing area is divided by the total labor force of the planting industry to construct the control variable *Scale*. Industrial agglomeration is closely related to economic development [66,67,68]. Therefore, this paper uses an agricultural location quotient to measure agricultural economic status, which is assigned to the variable *Status*. With increasing industrial agglomeration and industrialization, rural production and living equipment will be effectively improved, providing material conditions and product markets for agriculture and promoting high-quality development of the agricultural economy. Therefore, in this paper, the industrial added value of each region is used to measure the degree of industrialization of the region, with the variable being *Industrialization*. In addition, rural economic development is often affected by natural disasters [69]. Agricultural production performance is closely related to environmental quality. Any natural disasters can be devastating to farmers’ morale and may affect technological progress and efficiency [70], thus affecting the efficiency of agricultural carbon emissions reduction. Therefore, the degree of disaster measured by the affected crop area in each region is included as a control variable, *Damage*. Finally, moderating variables are chosen. Theoretically, mismatches in production materials directly inhibit output and distort the input decisions of micro subjects, resulting in loss of environmental welfare [53]. Therefore, this paper chooses farmer household land circulation and farmer household production equipment investment as the moderating variables, represented by *Ltr* and *Equipment*, respectively. Table 2 defines the variables used in the econometric model of this study. Except for proportional variables, all other variables were determined based on the data from 2010.

### 3.3. Econometric Model

This paper constructed the following econometric model to analyze the impact of broadband adoption on agricultural carbon emissions reduction efficiency by referring to Tang et al. [71].

(3) $$ACRE_{it}=\beta_{0}+\beta_{1}Broadband_{it}+\beta_{2}X_{it}+\lambda_{i}+\mu_{i}+\varepsilon_{it}$$

In Equation (3), $ACRE_{it}$ represents agricultural CO_2_ reduction efficiency in each province per year, and $Broadband_{it}$ represents the rural broadband use in province *i* as a proportion of time *t*. $\lambda_{i}$ is the fixed effects, $\mu_{i}$ is the time-fixed effects, and $\varepsilon_{it}$ is the random perturbation terms.

In order to analyze the moderating effect, this paper adds two moderating variables, land transfer (*Ltr*) and equipment investment (*Equipment*), based on the above benchmark model. The specific model is constructed as follows:

(4) $$ACRE_{it}=\beta_{0}+\beta_{1}Broadband_{it}+\beta_{2}Lcr_{it}+\beta_{3}Broadband_{it}\cdot Lcr_{it}+\beta_{4}X_{it}+\lambda_{i}+\mu_{i}+\varepsilon_{it}$$

(5) $$ACRE_{it}=\beta_{0}+\beta_{1}Broadband_{it}+\beta_{2}Equipment_{it}+\beta_{3}Broadband_{it}\cdot Equipment_{it}+\beta_{4}X_{it}+\lambda_{i}+\mu_{i}+\varepsilon_{it}$$

Equations (4) and (5) are the measurement models of *Ltr* and *Equipment*, respectively. Other variables are consistent with the benchmark model of Equation (3).

## 4. Results

### 4.1. Descriptive Statistics

Descriptive statistical results of variables are shown in Table 3. The maximum and minimum values of ACRE were 1.2 and 0.77, respectively. This shows that there is a certain gap in the efficiency of agricultural carbon emissions reduction in all provinces of China. The average broadband adoption rate was 22% and the minimum was 8.3%. According to the *Communication Industry Statistical Bulletin*, the proportion of fixed broadband access users in 2019 was 30%, an increase of 1.2 percentage points from the end of the previous year. Therefore, there remains much room for improvement in rural broadband access. The minimum number of computers per 100 households in rural areas was about 4 and the maximum number was about 75. This indicates a certain regional gap in the terminal application of rural broadband.

**Figure 2:**
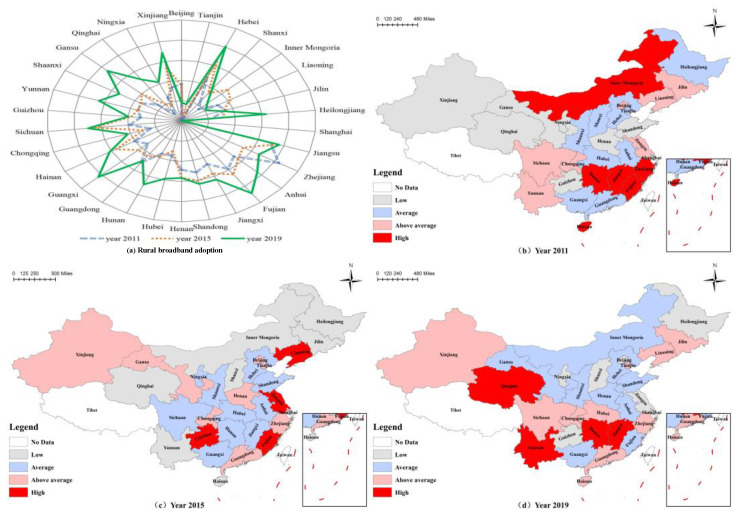
Spatial distribution of provincial rural broadband adaption and ACRE. Notes: There are four labels: (**a**–**d**).

In Figure 2, there are four labels: (a), (b), (c), and (d). First, label (a) shows the rural broadband adoption rate of 30 provinces in 2011, 2015, and 2019. Over time, the broadband adoption rate demonstrated a divergent outward rise. Second, labels (b) to (d) show the spatial distribution of ACER in 2011, 2015, and 2019. In the spatial map, darker colors indicate higher ARCE and lighter colors indicate lower ARCE. The pattern of ARCE values across regions shows a gradual shift of high ACRE from the eastern and central regions to the central and western regions. In 2011, the regions with high ARCE were mainly the developed eastern coastal provinces and the large agricultural provinces in central China. Compared with 2011, ARCE in the central region represented by Jiangxi and Hunan and the western region represented by Yunnan and Sichuan was significantly higher than the national average in 2019, while that in the eastern coastal region was significantly lower than the national average. The main reason for the increase in carbon emission levels in western China is the rapid increase in high-carbon agricultural production activities in western China, and the relative decrease in eastern China. This is fitting because China has vigorously implemented the “Broadband Village” pilot project in western and central China since 2014. Specific provinces involved include Yunnan, Sichuan, Chongqing, Jiangxi, and Hunan.

### 4.2. Rural Broadband Adoption and ACRE

The purpose of this study was to investigate the impact of broadband adoption on agricultural carbon reduction efficiency. Table 4 shows the estimated effects of broadband adoption on ACRE and reports the core conclusions of this paper. Model (1) controls the individual dummy variables of provinces. The influence coefficient of the core independent variable (*Broadband*) was 0.201 and significant at 5%. *Income* had a significant negative impact on ACRE, that is, within a certain range of income, the income of rural residents significantly reduces the ACRE. However, *Income squared* had a significant positive impact on ACRE, such that when income exceeded a certain range, the increase in the income of rural residents was conducive to improving ACRE. This confirms the U-shaped relationship between rural residents’ income and ACRE, consistent with the EKC hypothesis. At the same time, this result preliminarily verifies **H1** and **H2**. Broadband adoption significantly promotes ACRE. Models (2) and (3) report the results after adding other control variables. It can be seen that *Broadband* had the same positive impact on agricultural carbon emission reduction efficiency as the previous model, and it is significant at 5%. This indicates that the results in Table 4 have high stability and further verify **H1**. This may be because broadband adoption improve ACRE by promoting technological innovation, alleviating the distortion of industrial structure, and improving the efficiency of resource allocation [72]. In this paper, this is mainly mediated by direct effects and propagation effects. First, broadband adoption influence the rational use of inputs of factors through precision agriculture, thus reducing their adverse impact on the environment [32]. Second, regulating human environmental behavior through propagation effects contributes to significantly reducing CO_2_ emissions [73]. Therefore, broadband adoption can promote green agricultural production and achieve a significant improvement in ACRE. In terms of the positive impact of broadband adoption on the agricultural environment, our results were consistent with those by Ma and Zheng [46].

In addition, the scale of operation significantly affected ACRE. Possible reasons are as follows. First, the transfer of surplus agricultural labor concentrates cultivated land in the hands of the operators, causing a scale effect. This improves agricultural output, which is conducive to improving the enthusiasm of farmers for production standardization. Second, it provides intensive space for the large-scale socialization of agricultural services. Fragmented farmland is not conducive to increasing the scale of services, while larger scale production promotes cost reductions for green agricultural services. Agricultural economic status had a significant negative effect on ACRE. The reasons may be as follows. Areas with high agricultural economic status are the main grain-producing areas. With the goal of ensuring grain output, the concentrated input leads to pollution. Compared with non-major grain-producing areas, agricultural production activities in major grain-producing areas are more concentrated, and agricultural non-point source pollution per unit space increases with increasing production scale, resulting in greater environmental pollution. The degree of industrialization also had a significant effect on ACRE. This may be due to the crowding out of agriculture by industry to some extent. The development of industry requires the expansion of land in a large area, causing huge resource pressure for the development of agriculture. The frequency of natural disasters also negatively impacted ACRE. This may be explained by direct effects on the expected output and an impaired ability of agricultural activity to sequester carbon. Natural disasters may also negatively affect the motivation of farmers, including for green agricultural production. Therefore, considering agricultural development and ecological environmental protection under the goal of food security is a mainstream pursuit for current agricultural development.

### 4.3. Robustness Check

In order to verify the accuracy of the conclusion, it is necessary to test the robustness of the conclusion and replace the main independent variables. The computer penetration rate of rural residents (number of computers per 100 persons in rural areas) was used to replace the ratio of broadband adoption. Table 5 reports the impact of rural computer penetration on ACRE. The influence coefficient of computer penetration rate on ACRE is positive and significant at the 10% level. Therefore, the robustness test verifies that a larger rate of broadband adoption is associated with a more beneficial ACRE.

### 4.4. Endogeneity Problem

The core of this study is the nexus between broadband adoption and ACRE. However, an underlying endogeneity problem may occur. On one hand, there may be a reverse causality. If ACRE is higher in one province, it will have a spillover effect on the rest of the country, leading to a larger proportion of broadband adoption in other provinces. On the other hand, there may be some missing variables or measurement errors. These problems all lead to the underlying endogeneity problem. In order to alleviate and solve this problem, this paper uses the independent variable of a single lagged period and the heteroscedasticity-based recognition strategy for further estimation.

#### 4.4.1. Independent Variable of One Lag Period

Referring to Yu et al. [74], this paper uses independent variables lagged by one period to solve the endogenous problems caused by mutual causality. The lagging broadband adoption has a close relationship with the current period and is not affected by the efficiency of ACRE in the current period. Therefore, we use lagged first-stage broadband adoption as an instrumental variable to solve the endogenous problem caused by the reverse causality effect. The results in Table 6 show that the coefficient of ACRE is positive when broadband adoption lags by a period. Compared with the baseline results in Table 4, the estimated coefficient for broadband adoption varies from 0.111 to 0.074 and is significant at the 5% level. This further demonstrates the robustness of our results. That is, broadband adoption plays an important role in promoting ACRE.

#### 4.4.2. Recognition Strategy Based on Heteroscedasticity

To solve the endogenous problem, the heteroscedasticity-based recognition strategy from Lewbel [75] is adopted in this paper. This method uses the high-order moments of the data to generate a set of internal instrumental variables to improve the estimation validity, especially when the external validity of the instrumental variables is difficult to guarantee. According to Lewbel [75], recognition is achieved under two assumptions. First, the first-stage regression is performed on the benchmark econometric model, and the error obtained is heteroscedastic, which can be confirmed by the Breusch heteroscedasticity test. Second, there are covariates of the first-order and second-order errors that are independent of the conditional covariance. Table 7 reports the results of the instrumental variables. The results show that the F statistic of the first stage is greater than 10, indicating that there is no weak tool problem in the instrumental variables selected in this paper. The *p*-value of the Sargen-Baseman test statistic is greater than 0.05, which shows that all instrumental variables are exogenous and valid. Based on the above tests, it is concluded that the influence coefficient of broadband adoption on ACRE is 0.563, which is significant at the level of 5%. These results again demonstrate that the adoption of broadband can promote ACRE.

### 4.5. Further Analysis

Through the robustness and endogeneity analyses above, the relationship between rural broadband adoption and agricultural carbon emission reduction efficiency has been verified. However, existing studies have shown that compared with small farmers, land transfer operators are more likely to accept and adopt low-carbon agricultural technologies [55]. Information technology allows agricultural facilities to better realize the precision management of farmland to achieve carbon emissions reduction. Sun and Kim [76] found that ICTs effectively reduced CO_2_ intensity and spatial heterogeneity. Therefore, this paper further analyzes the moderating effects of land transfer (*Ltr*) and equipment investment (*Equipment*) on ACRE under the effect of broadband adoption, including further analysis of possible heterogeneity.

#### 4.5.1. Moderating Effects

Table 8 reports the regression results of the two moderating effects. It can be seen from Model (8) that the influence coefficient of the interaction term (*Broadband•Ltr*) is greater than zero and achieves statistical significance at the 10% level. This means that a higher proportion of broadband adoption results in improved ARCE by increasing land transfer rate. This conclusion verifies **H3**. A possible explanation for this phenomenon is that land transfer promotes the possibility of scaling up operation, causing a scale effect and improving agricultural output. This is conducive to improving the enthusiasm of farmers for production standardization and also increasing investment in green technology to provide an economic basis for promoting green agricultural production.

The moderating effect of farmers’ investment is reported in model (9). The interaction (*Broadband•Equipment*) has an impact factor greater than zero and passes the significance test at 10%. Therefore, improvements in informatization level support greater farmers’ investment in production equipment, thus increasing ACRE. This conclusion verifies **H4**. This can be attributed to the increased accessibility to precision agriculture that is provided by broadband adoption. While equipment investment with low-level informatization cannot realize precision agriculture and therefore result in increased CO_2_ emissions, the adoption of broadband in rural areas facilitates greater precision agriculture, supporting yield growth and the rational use of inputs. Thus, precision agriculture supported by broadband adoption minimizes the adverse impact on the environment.

#### 4.5.2. Heterogeneity Analysis

As previously described by Ma et al. [77], the provinces studied in this paper were grouped into three regions: western, central, and eastern. The results of spatial heterogeneity analysis are reported in Table 9. In eastern and central, broadband adoption has a positive effect on ACRE, but there was no significant effect in the west. Therefore, the western region is already making full use of the benefits brought by broadband infrastructure to expand the application of ICTs in agricultural and rural areas.

## 5. Conclusions

This study investigated the impact of rural broadband penetration on ACRE. Our provincial-level panel data demonstrated that the maximum and minimum values of ACRE were 1.2 and 0.77, indicating that there is a certain gap in the efficiency of agricultural carbon emissions reduction in all provinces of China. Further, the average broadband adoption rate was 22% and the minimum was 8.3%. Therefore, there remains much room for improvement in rural broadband access. In addition, the pattern of ARCE values across regions shows a gradual shift of high ACRE from the eastern and central regions to the central and western regions, which is similar to the results of He et al. and Yan et al. [78,79]. The main reason is that the agricultural production activities with high carbon emissions in the western region increased rapidly, while those in the eastern region decreased in comparison.

By looking into the direct impact of rural broadband adoption on ACRE and its related moderating effect, this study has generated a rich set of empirical findings. Firstly, based on the EKC hypothesis and considering the role of broadband adoption, our econometric results confirm the hypothesis that rural broadband adoption has a positive role in promoting agricultural carbon emissions reduction, supporting that which was reported in the literature [32,45,46]. Then, through further analysis of the mediation effect of land transfer and farmers’ investment in production equipment on ACRE, we found that the positive effect of broadband adoption on ACRE was more obvious when land transfer rates are high. This conclusion verifies the hypothesis that land transfer reduces the distortion degree of the factor market and has a regulating effect on ACRE. When farmers invest more in production equipment, the positive effect of broadband adoption is obvious. Finally, there was spatial heterogeneity between rural broadband adoption and ACRE. In eastern and central China, broadband adoption had a positive effect on ACRE, while in western China, broadband adoption had no significant effect. In particular, the SBM model and GML index method were used to measure ACRE, and the carbon sink of agricultural production was taken as one of the expected outputs, which was often ignored in previous studies.

The above empirical findings have several policy implications. (1) China is vigorously implementing the “Digital Villages” strategy, and a significant quantity of broadband infrastructure investment is gradually meeting the requirements of rural production and life. Therefore, future work will expand the application of ICTs at the production end of rural areas, advocate the use of broadband to connect farmers with green production and management and improve the efficiency of resource utilization. (2) The government should consider environmental externalities when formulating income redistribution policies. Transferring the surplus rural labor force can increase the income of rural families and enhance awareness of rural environmental protection, to realize the “win-win” of environmental protection and economic development. (3) Improving the land transfer rate is beneficial to increase the investment of green technology. At the same time, governments should continue to increase investment in intelligent agricultural production equipment. On the basis of the existing broadband infrastructure in rural areas, we should vigorously promote the use of information agricultural production equipment that is suitable for agricultural production, convenient for farmers, low cost, and simple to operate. (4) Rural broadband construction has lagged behind urban areas for a long time [80], so extra focus should be given to the promotion of household broadband penetration in developing areas. These areas can make good use of the Internet to improve energy efficiency and reduce the use of straw and coal in rural areas to reduce CO_2_ emissions.

Finally, it should be acknowledged that this work has some limitations. First, due to the lack of relevant data, our study only used broadband penetration to measure the application of ICTs development. To be more comprehensive, the application of ICTs development is measured in terms of infrastructure construction and utilization of ICTs. Therefore, in the following research, we will add indicators such as “network speed” and “Internet usage duration” to construct a comprehensive measurement of ICTs level. Second, this study investigated the impact of the application of ICTs on ACRE from a macro regional perspective. To further analyze the impact of the application of ICTs on the behavior of enterprises and farmers from a micro perspective, more representative samples should be used in future assessments of micro subjects’ behavior.

## Figures and Tables

**Table 1:** Agricultural carbon reduction efficiency measurement index system.

Type	Indicator Abbreviations	Indicator Index
Input indicators	Labor input	Number of First-born Employees (10,000 persons)
	Land input	Agricultural Sown Area (1000 Ha)
	Mechanical input	Total power of agricultural machinery (10,000 kW)
	Fertilizer input	Application amount of agricultural chemical fertilizer (10,000 tons)
	Pesticide input	Pesticide usage (10,000 tons)
	Plastic film input	Plastic film usage (10,000 tons)
	Irrigation input	Effective irrigation Area (1000 Ha)
Output indicators	Desirable output	Gross output value of Agriculture, Forestry, Animal Husbandry and Fishery (100 million Yuan)
		Agricultural carbon sink (10,000 tons)
	Undesirable output	Agricultural CO_2_ Emissions (10,000 tons)

**Table 2:** Variables definition.

Variable Name	Symbol	Variable Definition
Agricultural carbon reduction efficiency	ACRE	Accumulated value of carbon reduction efficiency of regional agriculture
Rural broadband adoption	*Broadband*	Ratio of regional rural broadband users to regional total users
Rural household computer penetration rate	*Computer*	Rural household computer penetration rate, that is, per 100 households computer ownership (sets/100 persons)
rural disposable income	*Income*	Natural logarithm of rural per capita disposable income
rural disposable income square	*Income square*	Natural logarithm of rural per capita disposable income square
Operation scale	*Scale*	Sown area/total labor in planting industry (hectares/person)
Agricultural economic status	*Status*	Location quotient: The ratio of the agricultural output value to the national agricultural output value divided by the ratio of the gross product of the province to the national gross product
Industrial added value	*Industrialization*	Natural logarithm of industrial added value
Ratio of disaster	*Damage*	Ratio of disaster area to sown area
Land transfer	*Ltr*	Ratio of circulation area to household contracted arable land area
Equipment investment	*Equipment*	Ratio of production equipment investment to fixed assets investment of rural households

**Table 3:** Descriptive statistics.

Variable Symbol	N	Mean	SD	Min	Max
ACRE	270	1.00	0.06	0.77	1.20
*Broadband*	270	0.22	0.10	0.08	0.44
*Computer*	270	25.33	14.45	4.04	74.70
*income*	270	9.30	0.41	8.30	10
*income square*	270	87	7.60	68	108
*Scale*	270	4.20	2.40	0.53	15
*Status*	270	1.20	0.68	0.04	4.10
*Industrialization*	270	8.80	0.97	6.20	11
*Damage*	270	0.15	0.12	0.006	0.62
*Ltr*	270	0.31	0.16	0.03	0.87
*Equipment*	270	0.16	0.13	0.003	0.68

Note: 1. See Table 2 for definitions of the Explained variables; 2. One USD was about 6.65 Chinese yuan as of December 2010.

**Table 4:** Rural broadband adoption and agricultural carbon reduction efficiency.

	(1)	(2)	(3)
	ACRE	ACRE	ACRE
*Broadband*	0.201 **	0.111 **	0.210 **
	(2.30)	(2.30)	(2.42)
*Income*	−0.889 ***	−0.608 ***	−1.101 ***
	(−2.87)	(−3.12)	(−3.62)
*income square*	0.047 ***	0.032 ***	0.057 ***
	(2.84)	(3.01)	(3.51)
*Scale*		0.004 **	0.012
		(2.35)	(1.20)
*Status*		−0.020 ***	−0.003
		(−3.69)	(−0.25)
*Industrialization*		−0.005	−0.014
		(−1.39)	(−0.70)
*Damage*		−0.101 *	−0.137 *
		(−1.81)	(−1.73)
_Cons	5.163 ***	3.979 ***	6.358 ***
	(3.61)	(4.46)	(4.69)
N	270	270	270
Province FE	YES	NO	YES
Year FE	NO	NO	YES
R-squared	0.033	0.127	0.085

Note: 1. See Table 2 for definitions of the variables; 2. *** *p* < 0.01, ** *p* < 0.05, and * *p* < 0.1; 3. Robust *t*-statistics in parentheses.

**Table 5:** Robustness analysis: Replacing the main independent variable.

	(4)
	ACRE
Computer	0.011 *
	(1.92)
Other control variables	Control
_Cons	3.196 *
	(1.87)
N	270
Province FE	Yes
Year FE	NO
R-squared	0.054

Note: 1. See Table 2 for definitions of the variables and other control variables; 2. *** *p* < 0.01, ** *p* < 0.05, and * *p* < 0.1; 3. Robust *t*-statistics in parentheses.

**Table 6:** Endogeneity problem: The independent variable lag for one period.

	(5)	(6)
	ACRE	ACRE
*Broadband*	0.074 *	0.093 *
	(1.78)	(1.69)
*income*	−0.683 ***	−1.055 **
	(−3.23)	(−2.45)
*income square*	0.035 ***	0.054 **
	(3.09)	(2.31)
*Scale*	0.004 **	0.015
	(2.51)	(1.12)
*Status*	−0.020 ***	0.028
	(−4.40)	(1.40)
*Industrialization*	−0.003	−0.012
	(−0.76)	(−0.48)
*Damage*	−0.105 *	−0.146
	(−1.67)	(−1.59)
_Cons	4.339 ***	6.168 ***
	(4.48)	(3.17)
N	240	240
Province FE	Yes	No
Year FE	No	No
R-squared	0.061	0.021

Note: 1. See Table 2 for definitions of the variables; 2. *** *p* < 0.01, ** *p* < 0.05, and * *p* < 0.1; 3. Robust *t*-statistics in parentheses.

**Table 7:** Endogenous treatment: Lewbel [75].

	(7)
	ACRE
*Broadband*	0.563 **
	(2.20)
Other control variables	Control
_Cons	0.901 ***
	(6.04)
N	270
Province FE	Yes
Year FE	Yes
R-squared	0.792
First-Stage F-Statistic	12.399
Sargan Statistic	16.380
*p*-value	0.063

Note: 1. See Table 2 for definitions of the variables; 2. *** *p* < 0.01, ** *p* < 0.05, and * *p* < 0.1; 3. Robust *t*-statistics in parentheses.

**Table 8:** Moderating effect results.

	(8)		(9)
	ACRE		ACRE
*Broadband*	0.124 **	Broadband	0.181 **
	(2.13)		(1.85)
*Ltr*	0.019	Equipment	−0.096 **
	(0.92)		(−2.47)
*Broadband•Ltr*	0.387 *	Broadband•Equipment	0.475 *
	(2.01)		(1.68)
*income*	−0.810 ***	income	−0.637 ***
	(−3.72)		(−3.02)
*income square*	0.025 ***	income square	0.024 ***
	(2.71)		(2.95)
*Scale*	0.002	Scale	0.007
	(1.16)		(0.75)
*Status*	−0.003	Status	0.001
	(−0.21)		(0.47)
*Industrialization*	−0.017	Industrialization	−0.006
	(−0.81)		(−1.32)
*Damage*	−0.074 *	Damage	−0.149 *
	(−1.76)		(−1.98)
_Cons	5.916 ***	_Cons	6.036 ***
	(4.32)		(3.92)
N	270	N	270
Province FE	Yes	Province FE	Yes
Year FE	Yes	Year FE	Yes
R-squared	0.059	R-squared	0.137

Note: 1. See Table 2 for definitions of the variables; 2. ***p < 0.01, **p < 0.05, and *p < 0.1; 3. Robust t-statistics in parentheses.

**Table 9:** Heterogeneity analysis results.

	(Western)	(Central)	(Eastern)
	ACRE	ACRE	ACRE
*Broadband*	0.197	0.089 *	0.115 *
	(1.09)	(1.81)	(1.74)
Other control variables	Control	Control	Control
_Cons	3.936	4.663 ***	8.188 *
	(1.51)	(2.91)	(1.84)
N	81	108	81
Province FE	No	No	No
Year FE	Yes	Yes	Yes
R-squared	0.221	0.057	0.047

Note: 1. See Table 2 for definitions of the variables; 2. *** *p* < 0.01, ** *p* < 0.05, and * *p* < 0.1; 3. Robust *t*-statistics in parentheses.

## Data Availability

The study did not report any data.
